# Recurrent Lobar Hemorrhages and Multiple Cortical Superficial Siderosis in a Patient of Alzheimer's Disease With Homozygous APOE ε2 Allele Presenting Hypobetalipoproteinemia and Pathological Findings of ^18^F-THK5351 Positron Emission Tomography: A Case Report

**DOI:** 10.3389/fneur.2021.645625

**Published:** 2021-07-07

**Authors:** Masaki Ikeda, Koichi Okamoto, Keiji Suzuki, Eriko Takai, Hiroo Kasahara, Natsumi Furuta, Minori Furuta, Yuichi Tashiro, Chisato Shimizu, Shin Takatama, Isao Naito, Mie Sato, Yasujiro Sakai, Manabu Takahashi, Masakuni Amari, Masamitsu Takatama, Tetsuya Higuchi, Yoshito Tsushima, Hideaki Yokoo, Masahiko Kurabayashi, Shun Ishibashi, Kenji Ishii, Yoshio Ikeda

**Affiliations:** ^1^Division of General Education (Neurology), Faculty of Health & Medical Care, Saitama Medical University, Saitama, Japan; ^2^Department of Neurology, Geriatrics Research Institute and Hospital, Maebashi, Japan; ^3^Department of Neurology, Gunma University Graduate School of Medicine, Maebashi, Japan; ^4^Department of Pathology, Geriatrics Research Institute and Hospital, Maebashi, Japan; ^5^Department of Neurology, Mito Medical Center, Mito, Japan; ^6^Department of Neurosurgery, Geriatrics Research Institute and Hospital, Maebashi, Japan; ^7^Department of Anesthesiology, Geriatrics Research Institute and Hospital, Maebashi, Japan; ^8^Department of Cardiovascular Medicine, Gunma University Graduate School of Medicine, Maebashi, Japan; ^9^Department of Diagnostic Radiology and Nuclear Medicine, Gunma University Graduate School of Medicine, Maebashi, Japan; ^10^Division of Endocrinology and Metabolism, Department of Internal Medicine, Jichi Medical University, Tochigi, Japan; ^11^Department of Pathology, Gunma University Graduate School of Medicine, Maebashi, Japan; ^12^Team for Neuroimaging, Tokyo Metropolitan Institute of Gerontology, Tokyo, Japan

**Keywords:** Alzheimer's disease, apolipoprotein ϵ allele, cerebral amyloid angiopathy, cortical superficial siderosis, recurrent lobar brain hemorrhages, hypobetalipoproteinemia, ^18^F-THK5351 PET, ^11^C-PiB PET

## Abstract

In Alzheimer's disease, the apolipoprotein E gene *(APOE)* ε2 allele is a protective genetic factor, whereas the *APOE* ε4 allele is a genetic risk factor. However, both the *APOE* ε2 and the *APOE* ε4 alleles are genetic risk factors for lobar intracerebral hemorrhage. The reasons for the high prevalence of lobar intracerebral hemorrhage and the low prevalence of Alzheimer's disease with the *APOE* ε2 allele remains unknown. Here, we describe the case of a 79-year-old Japanese female with Alzheimer's disease, homozygous for the *APOE* ε2 allele. This patient presented with recurrent lobar hemorrhages and multiple cortical superficial siderosis. The findings on the ^11^C-labeled Pittsburgh Compound B-positron emission tomography (PET) were characteristic of Alzheimer's disease. ^18^F-THK5351 PET revealed that the accumulation of ^18^F-THK 5351 in the right pyramidal tract at the pontine level, the cerebral peduncle of the midbrain, and the internal capsule, reflecting the lesions of the previous lobar intracerebral hemorrhage in the right frontal lobe. Moreover, ^18^F-THK5351 accumulated in the bilateral globus pallidum, amygdala, caudate nuclei, and the substantia nigra of the midbrain, which were probably off-target reaction, by binding to monoamine oxidase B (MAO-B). ^18^F-THK5351 were also detected in the periphery of prior lobar hemorrhages and a cortical subarachnoid hemorrhage, as well as in some, but not all, areas affected by cortical siderosis. Besides, ^18^F-THK5351 retentions were observed in the bilateral medial temporal cortices and several cortical areas without cerebral amyloid angiopathy or prior hemorrhages, possibly where tau might accumulate. This is the first report of a patient with Alzheimer's disease, carrying homozygous *APOE* ε2 allele and presenting with recurrent lobar hemorrhages, multiple cortical superficial siderosis, and immunohistochemically vascular amyloid β. The ^18^F-THK5351 PET findings suggested MAO-B concentrated regions, astroglial activation, Waller degeneration of the pyramidal tract, neuroinflammation due to CAA related hemorrhages, and possible tau accumulation.

## Introduction

The apolipoprotein E gene *(APOE)* ε2 allele has an exceptionally low likelihood of causing Alzheimer's dementia in *APOE2* homozygotes (1) and, it protects against Alzheimer's disease (AD) (2). In contrast, the *APOE* ε4 allele is a genetic risk factor of AD (3). However, the *APOE* ε2 and the *APOE* ε4 alleles, responsible for causing severe alterations in the vascular wall structure causing cerebral amyloid angiopathy (CAA), are common genetic risk factors for lobar intracerebral hemorrhage (ICH) (4–7). The reason for the high prevalence of lobar ICH despite the very low prevalence of AD in individuals with the *APOE* ε2 allele remains unknown. ^18^F-THK5351, which was developed as a tau binding tracer (8, 9), has been shown to strongly bind to monoamine oxidase B (MAO-B) (10). Recently, in a patient of cerebral infarction, the off-target retentions of ^18^F-THK5351 revealed Wallerian degeneration of the pyramidal tract, basal ganglia, thalamus, and cerebral cortices (11). In this report, we describe a patient with AD who carried a homozygous *APOE* ε2 allele. This patient presented with recurrent lobar hemorrhages and multiple cortical superficial siderosis (cSS) with hypobetalipoproteinemia. Additionally, several ^18^F-THK5351 PET findings reflected an increase in the MAO-B, reactive astrogliosis, and Wallerian degeneration of the pyramidal tract due to lobar ICH, as well as the possible accumulation of tau, related to AD pathology, in cerebral cortical areas.

## Methods

### ^11^C-PiB PET and ^18^F-THK5351 PET Neuroimages

^11^C-labeled Pittsburgh Compound B positron emission tomography (^11^C-PiB PET) was performed at the Gunma University Hospital as described previously by the authors (12, 13). ^18^F-THK5351 PET examinations were performed at the Tokyo Metropolitan Institute of Gerontology using the Discovery PET/CT 710 scanner (GE Healthcare, Milwaukee, WI, USA). Emission data were acquired for 20 min, starting 40 min after intravenous injection of ^18^F-THK5351 at a dose of approximately 190 MBq (5.1 mCi). In total, 47 slice images, with a voxel size of 2 × 2 × 3.27 mm and a matrix size of 128 × 128, were obtained. The data, collected in three-dimensional mode, were reconstructed after correcting for decay, attenuation, and scatter. ^18^F-THK5351 PET images were then normalized using the cerebellum as a reference region; cerebellar uptake was set to the value of one, as in the previously reported method (11, 14).

### Analyses of the Cerebrospinal Fluid (CSF)

A Cerebrospinal fluid was obtained by a lumbar puncture of the L3/L4 or L4/L5 intervertebral space, and the CSF samples were centrifuged for 10 min at 1,800 × g at 4°C within 3 h of collection. Samples were divided into aliquots of 0.5 mL and stored at −80°C in polypropylene tubes until they were analyzed using enzyme-linked immunosorbent assay (ELISA) kits for human CSF amyloid β (Aβ) Aβ1-42 and Aβ1-40 (FUJIFILM Wako Pure Chemical Corporation, Osaka, Japan) (12). Phosphorylated tau 181 (P-Tau) and human total tau (T-Tau) levels in the CSF were measured using the sandwich using ELISA INNOTEST^Ⓡ^ PHOSPHO-TAU (181P) (15) and the sandwich ELISA INNOTEST^Ⓡ^ T-Tau-Ag (both FUJIREBIO, Ghent, Belgium) (16), respectively, as described previously (12, 17).

### Pathological Examinations

Brain tissue samples were obtained when the patient underwent surgery for hematoma. Immunohistochemical studies were performed on using an anti-Aβ1-42 antibody and an anti-Aβ1-40 antibody (both IBL, Fujioka, Gunma, Japan).

### *APOE* Genotyping and Apolipoprotein E Protein Analyses

After obtaining informed consent for the genetic test of the *APOE* gene, we purified genomic DNA from lymphocytes in the peripheral blood of the patient. For the analysis of *APOE* allele polymorphism, purified genomic DNA samples were examined as previously described (12). The Apolipoprotein E protein was confirmed by the LSI Medience Corporation (Tokyo, Japan).

## Case Presentation and Results

**Figure 1:**
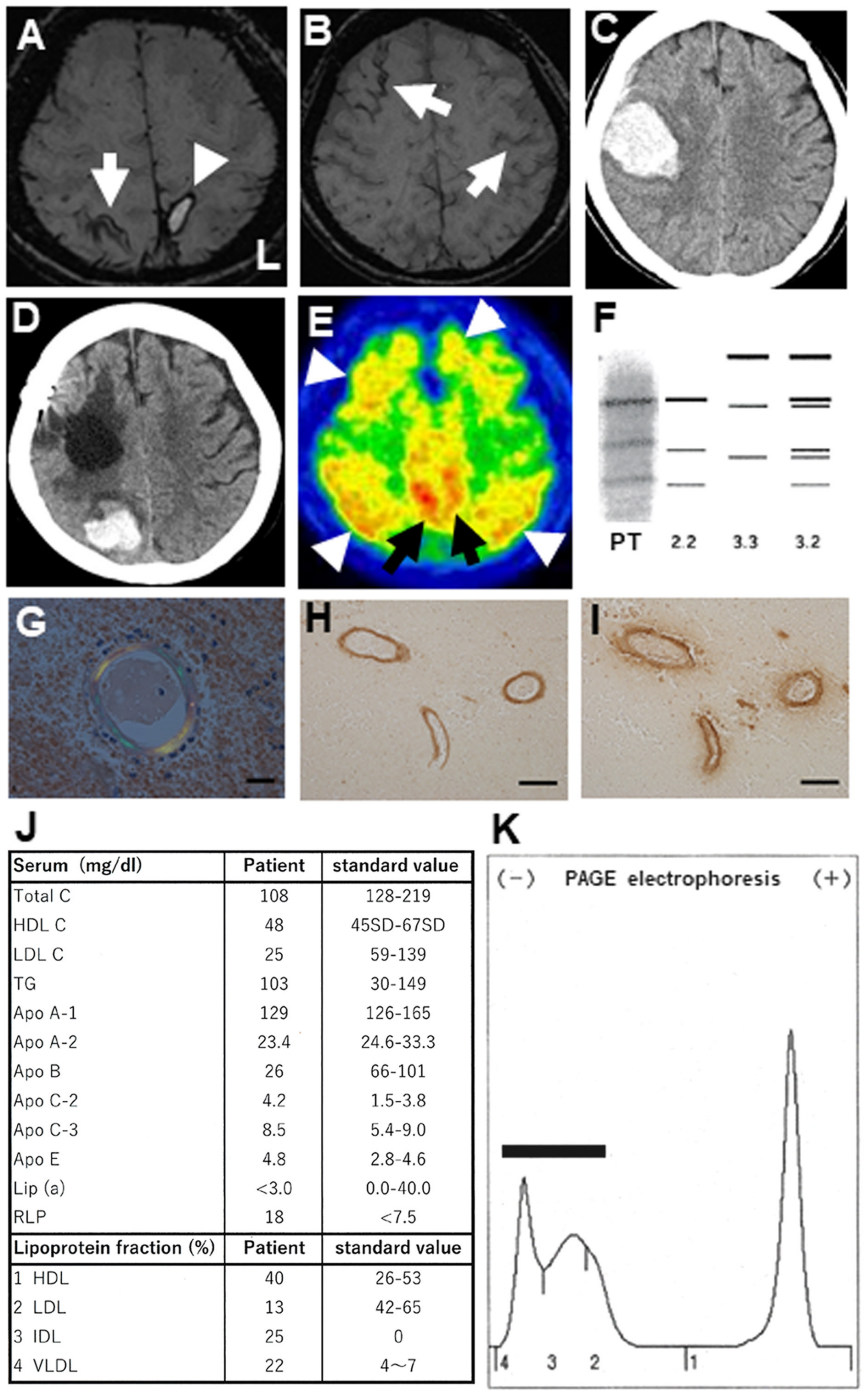
**(A)** Brain magnetic resonance imaging (MRI; 3T System, Skyra, Siemens, Germany) at the first admission. Susceptibility weighted images (SWI) showing a cortical hemorrhage (arrowhead) at the left parietal lobe cortex and cortical superficial siderosis (cSS) in the right parietal cortex (arrow). **(B)** In the same MRI, cSS was additionally observed in the left and right frontal cortices (arrows). **(C)** Brain computed tomography (CT) showing a second lobar hemorrhage at the right frontal subcortical area concurrent with left hemiparesis. **(D)** Brain CT showing a third lobar hemorrhage at the right parieto-occipital subcortical region. **(E)** ^11^C-PiB PET revealing widespread ^11^C-PiB retentions in the cerebral cortices (arrowheads) and precuneus (arrows). **(F)** Electrophoresis revealing a 2/2 pattern in the apolipoprotein E phenotype for this patient (PT). **(G)** Congophilic amyloid deposits shown in blood vessels of the resected brain tissue, as shown using polarizing microscopy (Scale bar: 20 μm). **(H,I)** Aβ-reactive structures in blood vessel walls identified by immunohistochemistry using anti-Aβ1-42 **(H)** and anti-Aβ1-40 **(I)** antibodies. Scale bars: 100 μm. **(J)** Laboratory serum results from the patient's first admission. **(K)** Serum lipoprotein electrophoresis reveals a broad-β pattern (bar), where intermediate-density lipoprotein (fraction 3) and very-low-density lipoprotein (fraction 4) are aberrantly elevated.

This report describes the case of a 79-year-old Japanese woman diagnosed with AD, presenting with recurrent hemorrhages despite the absence of vascular risk factors such as hypertension, diabetes mellitus, hypercholesterolemia, coronary heart disease, and smoking. However, fatty liver and hypocholesterolemia were observed on medical examination. Although her mother had died of a subarachnoid hemorrhage (SAH). At age 70 years, the patient experienced a loss of recent memory, and since then, the amnesia has gradually progressed. At the age of 72 years, she was admitted to the Gunma University Hospital for a headache caused by a brain hemorrhage; she felt a little headache and dullness without nausea, vomiting or loss of consciousness, a state that were quite different from a typical acute SAH. At the time of her first admission, cerebral magnetic resonance imaging (MRI) revealed a hemorrhage in the left parietal subcortical area (Figure 1A) and multiple regions of cSS (Figures 1A,B). Although there were no white matter hyperintensities, perivascular spaces, chronic ischemic infarcts, or lacunae, there were multiple regions of cSS and a few lobar microbleeds. On neuropsychological examinations, the scores were 23/30 in the Mini-Mental State Examination (MMSE) (disorientation of time, delayed recall deficits, acalculia, and agraphia), 19/30 in the Montreal Cognitive Assessment (MoCA) (visual-spatial disorientation, clock drawing, delayed recall, and disorientation of time), and 9/18 in the Frontal Assessment Battery (FAB) (deficits of similarities, motor series, conflicting instructions, and prehension behavior). The Instrumental Activities of Daily Living scales (IADL) (18) was 4/8 (Abilities required for shopping, food preparation, responsibility for own medications, and handling finances were disturbed). She could do activities of daily living (ADL) to some extent but needed some assistance from her family. According to gradual amnestic progression and executive dysfunction of ADL, she was diagnosed with mild dementia with AD at the first admission; the findings were also suggestive of possible CAA according to the modified Boston criteria (19). At age 73 years, the second cerebral hemorrhage occurred. This time she was admitted to the Geriatric Research Institute and Hospital for a left hemiparalysis with a lobar ICH at the right frontal lobe (Figure 1C). The hematoma was removed by a neurosurgical operation. The score of MMSE at the time of the second admission had decreased to 18/30, implying a cognitive decline, when compared to the first admission. The score of the revised Hasegawa Dementia Scale (HDS-R) (20) was 17/30, suggesting moderate dementia. At age 77 years, she was admitted for a headache for the third time; this time she had left hemispatial neglect, caused by a lobar ICH in the right parieto-occipital lobes (Figure 1D). Currently, she has no additional hemorrhages but is still affected by dementia and left hemiparalysis. The patient has not on any anti-platelet, anti-coagulation, or antihyperlipidemic drugs for the clinical course.

At the time of the first hospitalization, ^11^C-PiB PET revealed widespread ^11^C-PiB retentions in the cerebral cortices and precuneus, which were compatible with those of AD (Figure 1E).

The *APOE* genotyping revealed a homozygous ε2 allele (not shown). The apolipoprotein E (ApoE) was confirmed as a 2/2 protein by the western blot (Figure 1F). Congophilic amyloid deposits in blood vessels were confirmed on polarizing microscopy, undertaken using tissue resected during the second hospitalization (Figure 1G). Aβ-immunoreactive structures in blood vessels walls were revealed by an anti-Aβ1-42 antibody (Figure 1H) and an anti-Aβ1-40 antibody (Figure 1I) on immunohistochemical studies. Based on the pathological results, this case was suggestive of “probable CAA” with supporting pathological evidence according to the modified Boston criteria (19). Laboratory examination of the serum at the first admission demonstrated hypolipidemia, defined by low serum levels of total cholesterol (Total C), low-density lipoprotein cholesterol (LDL C), apolipoprotein A-2 (ApoA-2), and apolipoprotein B (Apo B). Chylomicrons were scarcely detectable (data not shown). The serum levels of apolipoprotein C-2 (ApoC-2), apolipoprotein E (ApoE), remnant-like lipoprotein (RLP), intermediate-density lipoprotein (IDL), and very-low-density lipoprotein (VLDL) were increased (Figure 1J). Furthermore, polyacrylamide gel (PAGE) electrophoresis of her serum revealed a broad-β band which seemed to be mimicking type III dysliplidemia (Figure 1K) (BML Co. Tokyo, Japan). There was no detection of aberrantly truncated apolipoprotein B on the western blot (data not shown). In the case, the CSF level of Aβ1-42 was 144.49 pg/mL, which was lower than that in non-dementia subjects (mean ± standard deviation, 431.55 ± 103.46 pg/mL, *n* = 20) and comparable to that in age-matched AD patients (183.32 ± 14.68 pg/mL, *n* = 25); the Aβ1-40 was 3037.22 pg/mL, which was lower than that in non-dementia subjects (mean ± standard deviation, 5118.79 ± 1882.47 pg/mL, *n* = 20) and age-matched AD patients (5358.47 ± 2376.80 pg/mL, *n* = 25); the P-Tau level was 42.94 pg/mL, which was higher than that in non-dementia subjects (29.33 ± 11.36 pg/mL, *n* = 20) and comparable to those of age-matched AD patients (75.40 ± 7.5 pg/mL, *n* = 25); and the T-Tau level was 424.50 pg/mL, which was higher than that in non-dementia subjects (143.55 ± 14.63 pg/mL, *n* = 20) and comparable to that in age-matched AD patients (503.30 ± 53.03 pg/mL, *n* = 25). These findings of CSF biomarkers and ^11^C-PiB PET of the patient were compatible with those of AD. Post-discharge the second hemorrhage at the age of 73 years, the 76-year-old patient was examined using ^18^F-THK5351 PET in a stable condition.

**Figure 2:**
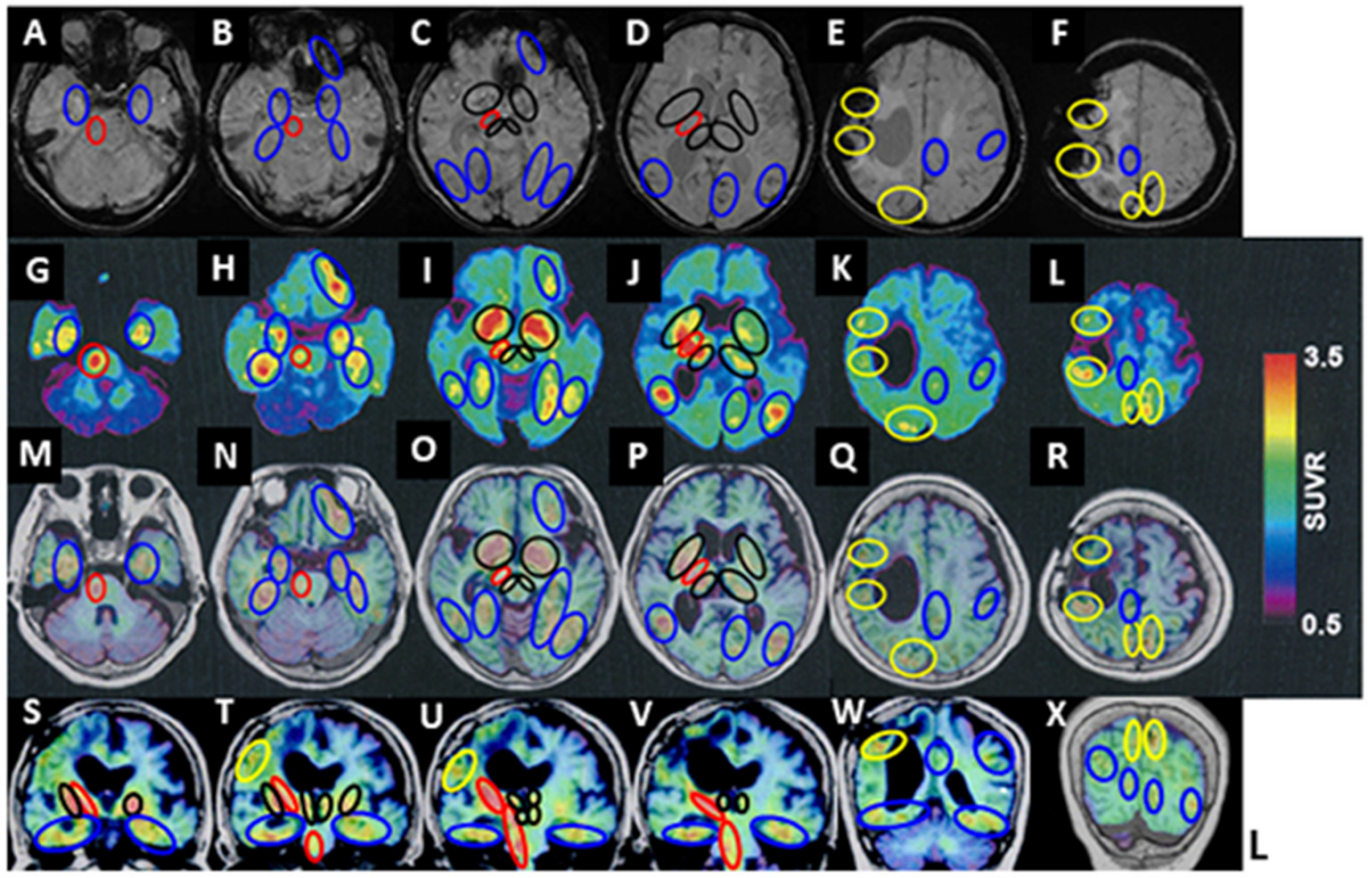
^18^F-THK5351 imaging and MRI of the patient's brain. **(A–F)** Susceptibility-weighted MRI. **(G–L)** ^18^F-THK5351. **(M–R)** ^18^F-THK 5351 on transverse MRI (T1-weighted images). **(S–X)** ^18^F-THK 5351 on coronal MRI (T1-weighted images). ^18^F-THK5351 retentions areas were observed in the pyramidal tract at the pontine level, the cerebral peduncle of the midbrain, and the internal capsule, all at the right side (red circles: **A-G-M-S**, **B-H-N-T**, **C-I-O-U**, and **D-J-P-V**). The bilateral substantia nigra of the midbrain, the globus pallidum, the anterior caudate nuclei, amygdala, and the medial areas of thalamus (**black circles**: **C-I-O-U** and **D-J-P-V**) were considered the off-target retentions of ^18^F-THK5351 binding to MAO-B. They were also visible along the previous lobar hemorrhagic lesions in the right frontal lobe and the left parietal cortex, as well as an asymptomatic old cortical SAH lesion in the right parietal cortex (yellow circles: **E-K-Q-W** and **F-L-R-X**). Additional ^18^F-THK5351 retentions were observed in the bilateral medial temporal areas (blue circles: **A-G-M-S** and **B-H-N-T**), the left anterior frontal cortical areas (blue circles: **B-H-N-T** and **C-I-O-U**), the posterior areas in bilateral temporal cortices, and the bilateral fusiform, lingual, and parahippocampal gyruses (blue circles: **C-I-O** and **D-J-P**).

^18^F-THK5351 retentions areas were observed at the right side of the pyramidal tract at the pontine level, the cerebral peduncle of the midbrain, and the internal capsule (red circles in Figure 2 **A-G-M-S, B-H-N-T, C-I-O-U**, and **D-J-P-V**). The off-target ^18^F-THK5351 retentions, binding to MAO-B derived from astrocytes, located in the pyramidal tract, were considered a result of a previous right frontal lobar hemorrhage.

In addition, they were detected at the bilateral substantia nigra of the midbrain, medial areas of the thalamus, the amygdala, the globus pallidum (**black circles** in Figure 2 **T, C-I-O-U, D-J-P-V**), and the anterior caudate nuclei (**black circles** in Figure 2 **D-J-P)**, in which originally contained a high concentration of MAO-B.

Besides, ^18^F-THK5351 retentions were visible along with previous lobar hemorrhagic lesions in the left parietal cortex (at the first hemorrhage) and the right frontal lobe (at the second hemorrhage), and an asymptomatic old cortical SAH lesion in the right parietal cortex (yellow circles in Figure 2 **E-K-Q-W** and **F-L-R-X**).

Additional ^18^F-THK5351 retentions were observed in the bilateral medial temporal areas (blue circles in Figure 2 **A-G-M-S** and **B-H-N-T**), the bilateral areas of entorhinal and parahippocampal gyruses, and hippocampus (blue circles in Figure 2 **B-H-N-T, C-I-O**), the bilateral areas of parahippocampal, fusiform, and lingual gyruses (blue circles in Figure 2 **C-I-O**), the left anterior frontal cortical areas (blue circles in Figure 2 **B-H-N** and **C-I-O**), the bilateral posterior areas in the temporal cortices (blue circles in Figure 2 **C-I-O** and D-J-P), bilateral basis areas in the temporal lobes (blue circles in Figure 2 **S, T, U, V**, and **W**), the left calcarine sulcus (blue circles in Figure 2 **D-J-P**), the left precuneus area and the left frontal cortex (blue circles in Figure 2 **E-K-Q-W**).

## Discussion

To our knowledge, this is the first report of an AD patient, homozygous for the *APOE* ε2 allele, presenting with recurrent lobar hemorrhage, vascular Aβ amyloid deposits, hypobetalipoproteinemia, and pathological ^18^F-THK5351 findings. The vasculopathologic changes associated with the *APOE* ε2 allele might have a crucial role in the severity and clinical course of lobar ICH, and the screening of patients with ICH for the *APOE* ε2 may identify those at increased risk of mortality and poor functional outcomes (21). The *APOE* ε2, as well as the *APOE* ε4, allele was reported to be significantly associated with lobar ICH and cSS (21, 22), and their presence may predict the recurrence of anti-coagulation-associated ICH (23). Clinically, some patients with the *APOE* ε2 allele present with hypocholesterolemia (24). In animal study, *APOE* ε2 transgenic mice showed a decreased serum level of low-density lipoprotein cholesterol (25). These biochemical aberrations may lead to the fragility in the structure of cerebral blood vessel walls, resulting in recurrent ICH in AD patients with the *APOE* ε2 allele (26, 27). *APOE* ε2/ε3 carriers have larger total volumes of white matter hyperintensities compared to *APOE* ε4 carriers and a higher prevalence of microbleeds compared to *APOE* ε3 homozygotes (28). Because the homozygous *APOE* ε2 allele has an extremely low frequency (1), no prior publication has confirmed a proven vascular amyloid pathology, in a homozygous *APOE* ε2 allele carrying AD patient with recurrent lobar ICH, using ^18^F-THK5351 PET and ^11^C-PiB PET. *In vivo* study revealed that the levels of Aβ40 and Aβ42 in both soluble and insoluble fractions of the brain lysate were reduced in *APOE* ε2, but not in *APOE* ε3 or *APOE* ε4 gene integrated in human amyloid precursor protein/human presenilin 1 (APP/PS1) double transgenic mice (29). The CAA patients had a distinctive CSF biomarker profile, with significantly lower concentrations of Aβ40 and Aβ42 than those of non-demented controls and AD patients, which were found in *APOE* ε2, ε4, and ε3 genotypes (30–33). Lower CSF levels of Aβ1-40 and Aβ1-42 in an AD patient with CAA carrying *APOE* ε2 homozygote had not been reported before this case. So far, pathological reports of autopsy studies showed reduced neurofibrillary tangles (NFTs) in postmortem AD brains of *APOE* ε2 carriers (2, 34, 35). Although the mechanism underlying this reduction of NFTs is poorly understood; as *APOE* ε2 was significantly correlated with tau pathology only in Aβ positive AD patients but not with those in Aβ negative individuals, the protective effect of *APOE* ε2 against AD tau may be partially mediated through its effect on Aβ deposition (36). It remains still controversial why *APOE* ε2 may protect against tau pathology independently of Aβ in AD (37–39). ^18^F-THK5351 was initially developed to target tau aggregates in NFTs (8, 9), however, ^18^F-THK5351 has been shown to strongly bind off-target to MAO-B in the basal ganglia and thalamus (10), as well as the melanin-containing cells of the substantia nigra (40). ^18^F-THK5351 is also highly concentrated in astrocytes, compared to other types of glial cells (41, 42). The MAO-B concentration is also increased in reactive astrogliosis (43), which describes a spectrum of astrocytic changes that occur in response to brain injury (44). On the other hand, Wallerian degeneration, the progressive anterograde demyelination and disintegration of distal axons, develops following a brain injury to neuronal soma or proximal axons (45). In the early stage of brain injury, axonal swelling, and breakdown of myelin sheath occur, followed by loss of myelinated fibers and infiltration of macrophages. In the later stage, the degenerated axons are replaced by gliosis, which is primarily the proliferation of astrocytes to form a scar. A previous case report demonstrated ipsilateral myelin loss and gliosis in the pyramidal tract lesion 5 years after a left middle cerebral artery infarction (45). As for the other tau PET tracers and CAA, two reports of Tau PET have been published (46, 47). In the former report, paired helical filament (PHF) tau burden overlapped cerebral microbleeds and/or cSS on ^18^F-AV1451 PET, raising the possibility that the characteristic markers of vascular amyloid were associated with local production of PHF tau (46). In the latter report, the amyloid deposition, evaluated by ^18^F-florbetaben PET, was not different in vicinity of cSS, but tau depositions were elevated in vicinity of cSS-affected regions compared to non-cSS-affected brain regions. Their case of probable CAA suggested that cSS may be associated with an elevation in local tau accumulation but not with an increase in fibrillary amyloid deposition (47). ^18^F-THK5351 PET has been reported to strongly bind to MAO-B derived from astrocytes and possibly tau, probably reflecting astrogliosis related CAA hematoma and cSS, also possibly tau accumulation in AD pathology (48). In our case, 3 year have passed since the ^18^F-THK5351 PET examination for the previous ICH in the right frontal lobe presenting as contralateral hemiparalysis. In this patient, Wallerian degeneration may have developed in the ipsilateral pyramidal tract followed by astrogliosis, and the marker ^18^F-THK5351 PET, targeting MAO-B containing tissues, may have identified this Wallerian degeneration. The ^18^F-THK5351 is now recognized as a dual-purpose compound that binds to both MAO-B and tau aggregates, thus tau isoforms might have accumulated in the damaged fasciculus. MAO-B is highly concentrated in astrocytes and serotonergic and histaminergic neurons (43), astrocytes proliferate in response to inflammation caused by brain injury (44). Therefore, regional changes in MAO-B concentration can be an index of astrogliosis and be detected on ^18^F-THK5351 PET imaging, although tau accumulation may co-exist in the AD brain (48). These findings suggest that ^18^F-THK5351 accumulates in lesions where astrogliosis occurs and that ^18^F-THK5351 PET can be an imaging modality to visualize and quantify astrogliosis, as recently demonstrated in patients with a cerebral infarction or neurological disorders (49–51). In our case, ^18^F-THK5351 retentions were observed in some, but not all, regions with cSS, presumably because this effect might depend on severity of cSS and the degree of astrogliosis that is correlated with the extent of hemosiderin deposition (52). ^18^F-THK5351 retentions were observed only in some areas rather than all peripheral regions at the lobar hemorrhage in this patient. Several cSS lesions reflected ^18^F-THK5351 PET retentions, however, the other cSS lesions did not reflect, which might probably depend upon severity of cSS or neuroinflammation of CAA. It seems that old cSS showed less or no THK5351 retention [such as in bilateral prefrontal lobes cortices/subcortices (Figure 2 **D-J-P and E-K-Q**)], while the recent lesions of hematoma or siderosis showed THK5351 retentions [such as in left parietal cortical area (hematoma at the first admission) and right frontal cortico-subcortical areas (hematoma at the second admission)]. This case signifies that both AD and CAA pathologies could co-occur and contribute to cognitive decline (53), indicating that CAA, especially, cSS strongly increases the risk of hemorrhages, even when the clinical changes are gradual. We found that CSF Aβ1-40 of this patient was lower than those of AD patients and non-demented subjects, as, in the recent publication, the levels of CSF Aβ1-40 in AD with disseminated cSS were lower than that in AD patients with focal cSS and those without cSS (54, 55). This result is thought be in accordance with neuropathological reports demonstrating deposition of Aβ40 within the vessel walls of CAA patients (56). CSF P-Tau levels, in this case, were not significantly higher than those in AD patients, because CSF P-Tau levels in AD patients with CAA pathology have been reported to be either slightly elevated or within the normal levels (55, 57, 58). This could be because of the change in permeability of the cerebral blood vessels due to CAA; decreased ability of parenchymal P-Tau to flow out into the lumen of vessels or less amount of tau phosphorylation in CAA than in AD patients may have led to lower P-Tau levels in CSF. On the other hand, Aβ 40 is thought to deposit in the blood vessels affected by CAA, rather than flowing into the lumen of vessels (59). Although the fact that the pathophysiologic mechanisms are not completely understood, recent studies show that *APOE2* allele is strongly associated with cSS and increased frequency and severity of lobar ICH (22). Furthermore, while *APOE* ε2 promotes so-called CAA-related vasculopathic changes (vessel cracking, detachment, and delamination of the outermost layer of the tunica media, and fibrinoid necrosis), this can lead to vessel rupture (5, 22), another evidence of increased ICH risk in *APOE* ε2 carriers was recently reported, with or without AD (60). Hypobetalipoproteinemia may not be a direct cause for the ICH in this case, but might be an additional contributing factor, because hypocholesterolemia was reported to be a risk factor of ICH (61–63). This AD case carried the homozygous *APOE* ε2 allele and severe hypocholesterolemia, which may have exacerbated vascular changes in the CAA, thereby leading to more severe CAA, multiple cSS, and recurrent lobar ICH. The mechanism underlying the risk for ICH because of an *APOE* ε2 genotype is still unclear and perhaps complex (60), which should be elucidated hereafter. In future, a large-scale investigation of cholesterol levels in AD patients with a homozygous *APOE* ε2 allele will be required to define its clinical significance and etiologies. This AD case with multiple cSS regions and lobar hemorrhages is also worth reporting; it demonstrates that ^18^F-THK5351 retentions reflected MAO-B, reactive astrogliosis, and Wallerian degeneration of the pyramidal tract due to CAA related lobar ICH, and possibly tau accumulation.

Finally, the therapies targeting *APOE* ε2 and APOE2 for AD have been extensively developed; a viral-mediated *APOE* ε2 overexpression, converting *APOE* ε4 to *APOE* ε2, and plasma APOE-based therapy were described detail in a recent review (64). Although these trials have some effect, unfortunately, they have not been effective, regardless of their successful experimental results. Besides severe CAA pathology, this case presented with hypobetalipoproteinemia, however, we could not elucidate the clear molecular and pathological relationships between them. To validate the mechanisms of effective therapies targeting APOE2/*APOE* ε2, more evidence from humans and animal models is required. Further, more AD patients carrying the *APOE* ε2 allele are required to analyze pathological and biochemical findings. Furthermore, improved understanding of the roles of APOE2/*APOE* ε2 in other diseases, CAA/Stroke and different proteinopathies, including tau, TDP-43, and α-synuclein pathologies, may allow a comprehensive assessment of the safety of APOE2 targeted therapeutics for AD.

A limitation of this study is that it includes only one AD patient carrying a homozygous *APOE* ε2 allele with recurrent hemorrhages and multiple cSS regions. This impedes statistical analyses for cholesterol levels in comparison to other *APOE* alleles or healthy control groups. The other limitation is that we could not confirm how histopathological association between ^18^F-THK5351 retention and astrogliosis pathology since we histologically observed only a part of the brain. To better understand the pathological mechanism, a detailed investigation using neuroradiological, biochemical, genetical, and pathological methods is required in CAA and AD patients.

## Data Availability Statement

The raw data supporting the conclusions of this manuscript will be made available by the authors, without undue reservation, to any qualified researcher.

## Ethics Statement

The studies involving human participants were reviewed and approved by the ethics committee of the Gunma University Hospital. The patients/participants provided their written informed consent to participate in this study. Written informed consent was obtained from the individual(s) for the publication of any potentially identifiable images or data included in this article. Written consent to publish the clinical information was obtained from the patient's family.

## Author Contributions

MI collected the clinical data, interpreted the data, and wrote the manuscript. ET analyzed genomic DNA from the patient's blood samples and CSF biomarkers from the patient. KO, KS, and HY performed pathological examinations and evaluation of the results. TH and YTs performed the neuroimaging examination for ^11^C PiB-PET, and KI performed and evaluated the neuroimaging examination for ^18^F THK5351-PET. MF, NF, YTa, CS, MS, YS, MA, and MTakat collected the clinical information. SI, MK, and MTakah performed and evaluated the western blot of ApoB protein, also discussed the hypobetalipoproteinemia in this patient. ST and IN performed the neurosurgical operation to removal the hematoma at the second admission. MI performed the clinical data analysis and evaluated the specificities and neurological significances. All authors contributed to the article and approved the submitted version.

## Conflict of Interest

The authors declare that the research was conducted in the absence of any commercial or financial relationships that could be construed as a potential conflict of interest.

## Abbreviations

*APOE*: apolipoprotein E gene
AD: Alzheimer's disease
ICH: intracerebral hemorrhage
CAA: cerebral amyloid angiopathy
MAO-B: monoamine oxidase B
cSS: cortical superficial siderosis
^11^C PiB-PET: ^11^C-labeled Pittsburgh Compound B-positron emission tomography
Aβ: amyloid β
P-Tau: phosphorylated tau 181
T-Tau: human total tau
CSF: cerebrospinal fluid
SAH: subarachnoid hemorrhage
MRI: magnetic resonance imaging
WMH: white matter hyperintensities
PVC: perivascular spaces
ADL: activities of daily living.

## References

[1] Reiman EM Arboleda-Velasquez JF Quiroz YT Huentelman MJ Beach TG Caselli RJ. Exceptionally low likelihood of Alzheimer's dementia in APOE2 homozygotes from a 5,000-person neuropathological study. Nat Commun. (2020) 11:667. 10.1038/s41467-019-14279-8 32015339 PMC6997393

[2] Serrano-Pozo A Qian J Monsell S Betensky R Hyman B. APOEε2 is associated with milder clinical and pathological Alzheimer's disease. Ann Neurol. (2015) 77:917–29. 10.1002/ana.24369 25623662 PMC4447539

[3] Corder EH Saunders AM Strittmatter WJ Schmechel DE Gaskell PC Small G. Gene dose of apolipoprotein E type 4 allele and the risk of Alzheimer's disease in late onset families. Science. (1993) 261:921–3. 10.1126/science.8346443 8346443

[4] Nicoll JA Burnett C Love S Graham DI Dewar D Ironside JW. High frequency of apolipoprotein E ε2 allele in hemorrhage due to cerebral amyloid angiopathy. Ann Neurol. (1997) 41:716–21. 10.1002/ana.410410607 9189032

[5] Greenberg SM Vonsattel JP Segal AZ Chiu RI Clatworthy AE Liao A. Association of apolipoprotein E ε2 and vasculopathy in cerebral amyloid angiopathy. Neurology. (1998) 50:961–5. 10.1212/wnl.50.4.961 9566379

[6] O'Donnell HC Rosand J Knudsen KA Furie KL Segal AZ Chiu RI. Apolipoprotein E genotype and the risk of recurrent lobar intracerebral hemorrhage. N Eng J Med. (2000) 342:240–5. 10.1056/NEJM200001273420403 10648765

[7] Rannikmäe K Kalaria RN Greenberg SM Chui HC Schmitt FA Samarasekera N. APOE associations with severe CAA-associated vasculopathic changes: collaborative meta-analysis. J Neurol Neurosurg Psychiatry. (2014) 85:300–5. 10.1136/jnnp-2013-306485 24163429 PMC4018226

[8] Harada R Okamura N Furumoto S Furukawa K Ishiki A Tomita N. 18F-THK5351: a novel PET radiotracer for imaging neurofibrillary pathology in Alzheimer disease. J Nucl Med. (2016) 57:208–14. 10.2967/jnumed.115.164848 26541774

[9] Tago T Furumoto S Okamura N Harada R Adachi H Ishikawa Y. Structure-activity relationship of 2-arylquinolines as PET imaging tracers for tau pathology in Alzheimer disease. J Nucl Med. (2016) 57:608–14. 10.2967/jnumed.115.166652 26697966

[10] Ng KP Pascoal TA Mathotaarachchi S Therriault J Kang MS Shin M. Monoamine oxidase B inhibitor, selegiline, reduces ^18^F-THK5351 uptake in the human brain. Alzheimers Res Ther. (2017) 9:25. 10.1186/s13195-017-0253-y 28359327 PMC5374697

[11] Ishibashi K Kameyama M Tago T Toyohara J Ishii K. Potential use of 18F-THK5351 PET to identify wallerian degeneration of the pyramidal tract caused by cerebral infarction. Clin Nucl Med. (2017) 42:e523–4. 10.1097/RLU.0000000000001868 29076904

[12] Ikeda M Tashiro Y Takai E Kurose S Fugami N Tsuda K. CSF levels of Aβ1-38/Aβ1-40/Aβ1-42 and ^11^C-PiB PET studies in three clinical variants of primary progressive aphasia and Alzheimer's disease. Amyloid. (2014) 21:238–45. 10.3109/13506129.2014.949231 25139672

[13] Kasahara H Ikeda M Nagashima K Fujita Y Makioka K Tsukagoshi S. Deep white matter lesions are associated with early recognition of dementia in Alzheimer's disease. J Alzheimers Dis. (2019) 68:797–808. 10.3233/JAD-180939 30775989

[14] Ishibashi K Miura Y Hirata K Toyohara J Ishii. Relationship between the temporal course of astrogliosis and symptom improvement in cerebral infarction: report of a case monitored using ^18^ F-THK5351 positron emission tomography. BMC Med Imaging. (2020) 20:81. 10.1186/s12880-020-00481-4 32664871 PMC7362635

[15] Vanmechelen E Vanderstichele H Davidsson P Van Kerschaver E Van Der Perre B Sjögren M Andreasen N. Quantification of tau phosphorylated at threonine 181 in human cerebrospinal fluid: a sandwich ELISA with a synthetic phosphopeptide for standardization. Neurosci Lett. (2000) 285:49–52. 10.1016/s0304-3940(00)01036-3 10788705

[16] Andreasen N Minthon L Davidsson P Vanmechelen E Vanderstichele H Winblad B. Evaluation of CSF-tau and CSF-Aβ42 as diagnostic markers for Alzheimer's disease in clinical practice. Arch Neurol. (2001) 58:373–9. 10.1001/archneur.58.3.373 11255440

[17] Ikeda M Kuwabara T Takai E Kasahara H Furuta M Sekine A. Increased neurofilament light chain and YKL-40 CSF Levels in one Japanese IBMPFD patient with VCP R155C mutation: a clinical case report with CSF biomarker analyses. Front Neurol. (2020) 11:757. 10.3389/fneur.2020.00757 32849216 PMC7431878

[18] Lawton MP Brody EM. Assessment of older people: self-maintaining and instrumental activities of daily living. Gerontologist. (1969) 9:179–86. 5349366

[19] Linn J Halpin A Demaerel P Ruhland J Giese AD Dichgans M. Prevalence of superficial siderosis in patients with cerebral amyloid angiopathy. Neurology. (2010) 74:1346–50. 10.1212/WNL.0b013e3181dad605 20421578 PMC2875936

[20] Katoh S Shimogaki H Onodera A Ueda H Oikawa K Ikeda K. Development of the revised version of Hasegawa's Dementia-Scale (HDS-R). Jpn J Geriatr Psychiatry. (1991) 2:1339–47. (Japanese)

[21] Biffi A Anderson CD Jagiella JM Schmidt H Kissela B Hansen BM. APOE genotype and extent of bleeding and outcome in lobar intracerebral haemorrhage: a genetic association study. Lancet Neurol. (2011) 10:702–9. 10.1016/S1474-4422(11)70148-X 21741316 PMC3153411

[22] Charidimou A Zonneveld HI Shams S Kantarci K Shoamanesh A Hilal S. APOE and cortical superficial siderosis in CAA: meta-analysis and potential mechanisms. Neurology. (2019) 93:e358–71. 10.1212/WNL.0000000000007818 31243071 PMC6669935

[23] Biffi A Urday S Kubiszewski P Gilkerson L Sekar P Rodriguez-Torres A. Combining imaging and genetics to predict recurrence of anticoagulation-associated intracerebral hemorrhage. Stroke. (2020) 51:2153–60. 10.1161/STROKEAHA.120.028310 32517581 PMC7311289

[24] Mahley RW Huang Y Rall SC. Pathogenesis of type III hyperlipoproteinemia (dysbetalipoproteinemia): questions, quandaries, and paradoxes. J Lipid Res. (1999) 40:1933–49. 10552997

[25] Huang Y Liu XQ Rall SC Mahley RW. Apolipoprotein E2 reduces the low-density lipoprotein level in transgenic mice by impairing lipoprotein lipase-mediated lipolysis of triglyceride-rich lipoproteins. J Biol Chem. (1998) 273:17483–90. 10.1074/jbc.273.28.17483 9651338

[26] McCarron MO Nicoll JA Stewart J Ironside JW Mann DM Love S. The apolipoprotein E epsilon2 allele and the pathological features in cerebral amyloid angiopathy-related hemorrhage. J Neuropathol Exp Neurol. (1999) 58:711–8. 10.1097/00005072-199907000-00005 10411341

[27] Nelson PT Pious NM Jicha GA Wilcock DM Fardo DW Estus S. APOE-ε2 and APOE-ε4 correlate with increased amyloid accumulation in cerebral vasculature. J Neuropathol Exp Neurol. (2013) 72:708–15. 10.1097/NEN.0b013e31829a25b9 23771217 PMC3715146

[28] Groot C Sudre CH Barkhof F Teunissen CE van Berckel BN Seo SW. Clinical phenotype, atrophy, and small vessel disease in APOEε2 carriers with Alzheimer disease. Neurology. (2018) 91:e1851–9. 10.1212/WNL.0000000000006503 30341156

[29] Hudry E Dashkoff J Roe AD Takeda S Koffie RM Hashimoto T. Gene transfer of human ApoE isoforms results in differential modulation of amyloid deposition and neurotoxicity in mouse brain. Sci Transl Med. (2013) 5: 212ra161. 10.1126/scitranslmed.3007000 24259049 PMC4334150

[30] Costa AS Pinho J Kučikiene D Reich A Schulz JB Reetz K. Cerebral amyloid angiopathy in amyloid-positive patients from a memory clinic cohort. J Alzheimers Dis. (2021) 79:1661–72. 10.3233/JAD-201218 33492291

[31] Banerjee G Ambler G Keshavan A Paterson RW Foiani MS Toombs J. Cerebrospinal fluid biomarkers in cerebral amyloid angiopathy. J Alzheimers Dis. (2020) 74:1189–1201. 10.3233/JAD-191254 32176643 PMC7242825

[32] Charidimou A Imaizumi T Moulin S Biffi A Samarasekera N Yakushiji Y. Brain hemorrhage recurrence, small vessel disease type, and cerebral microbleeds: a meta-analysis. Neurology. (2017) 89:820–29. 10.1212/WNL.0000000000004259 28747441 PMC5580863

[33] Shams S Martola J Charidimou A Cavallin L Granberg T Shams M. Cortical superficial siderosis: prevalence and biomarker profile in a memory clinic population. Neurology. (2016) 87:1110–7. 10.1212/WNL.0000000000003088 27534713 PMC5027806

[34] Bennett DA De Jager PL Leurgans SE Schneider JA. Neuropathologic intermediate phenotypes enhance association to Alzheimer susceptibility alleles. Neurology. (2009) 72:1495–503. 10.1212/WNL.0b013e3181a2e87d 19398704 PMC2677477

[35] Nagy Z Esiri MM Jobst KA Johnston C Litchfield S Sim E. Influence of the apolipoprotein E genotype on amyloid deposition and neurofibrillary tangle formation in Alzheimer's disease. Neuroscience. (1995) 69:757–61. 10.1016/0306-4522(95)00331-c 8596645

[36] Farfel JM Yu L De Jager PL Schneider JA Bennett DA. Association of APOE with tau-tangle pathology with and without β-amyloid. Neurobiol Aging. (2016) 37:19–25. 10.1016/j.neurobiolaging.2015.09.011 26481403 PMC4716785

[37] Goldberg TE Huey ED Devanand DP. Association of APOE ε2 genotype with Alzheimer's and non-Alzheimer's neurodegenerative pathologies. Nat Commun. (2020) 11:4727. 10.1038/s41467-020-18198-x 32948752 PMC7501268

[38] Zhao N Liu CC Van Ingelgom AJ Linares C Kurti A Knight JA. APOE ε2 is associated with increased tau pathology in primary tauopathy. Nat Commun. (2018) 9:4388. 10.1038/s41467-018-06783-0 30348994 PMC6197187

[39] Ghebremedhin E Schultz C Botez G Rüb U Sassin I Braak E. Argyrophilic grain disease is associated with apolipoprotein E epsilon 2 allele. Acta Neuropathol. (1998) 96:222–4. 10.1007/s004010050886 9754952

[40] Tago T Toyohara J Harada R Furumoto S Okamura N Kudo Y. Characterization of the binding of tau imaging ligands to melanin-containing cells: putative off-target-binding site. Ann Nucl Med. (2019) 33:375–82. 10.1007/s12149-019-01344-x 30796626

[41] Gulyás B Pavlova E Kása P Gulya K Bakota L Várszegi S. Activated MAO-B in the brain of Alzheimer patients, demonstrated by [^11^C]-L-deprenyl using whole hemisphere autoradiography. Neurochem Int. (2011) 58:60–8. 10.1016/j.neuint.2010.10.013 21075154

[42] Levitt P Pintar JE Breakefield XO. Immunocytochemical demonstration of monoamine oxidase B in brain astrocytes and serotonergic neurons. Proc Natl Acad Sci USA. (1982) 79:6385–9. 10.1073/pnas.79.20.6385 6755469 PMC347126

[43] Saura J Luque JM Cesura AM Da Prada M Chan-Palay V Huber G. Increased monoamine oxidase B activity in plaque-associated astrocytes of Alzheimer brains revealed by quantitative enzyme radioautography. Neuroscience. (1994) 62:15–30. 10.1016/0306-4522(94)90311-5 7816197

[44] Sofroniew MV. Molecular dissection of reactive astrogliosis and glial scar formation. Trends Neurosci. (2009) 32:638–47. 10.1016/j.tins.2009.08.002 19782411 PMC2787735

[45] Jones KC Hawkins C Armstrong D Deveber G Macgregor D Moharir M. Association between radiographic Wallerian degeneration and neuropathological changes post childhood stroke. Dev Med Child Neurol. (2013) 55:173–7. 10.1111/dmcn.12010 23171053

[46] Kim HJ Cho H Werring DJ Jang YK Kim YJ Lee JS. 18F-AV-1451 PET imaging in three patients with probable cerebral amyloid angiopathy. J Alzheimers Dis. (2017) 57:711–6. 10.3233/JAD-161139 28282808

[47] Brendel M Catak C Beyer L Linn J Wahl H Janowitz D. Colocalization of tau but not β-amyloid with cortical superficial siderosis in a case with probable CAA. Case Rep Neurol. (2020) 12:232–7. 10.1159/000506765 32774280 PMC7383156

[48] Harada R Ishiki A Kai H Sato N Furukawa K Furumoto S. Correlations of ^18^F-THK5351 PET with postmortem burden of tau and astrogliosis in Alzheimer disease. J Nucl Med. (2018) 59:671–4. 10.2967/jnumed.117.197426 28864633

[49] Ishibashi K Miura Y Hirata K Toyohara J Ishii K. ^18^F-THK5351 PET can identify astrogliosis in multiple sclerosis plaques. Clin Nucl Med. (2020) 45:e98–100. 10.1097/RLU.0000000000002751 31348091

[50] Schönecker S Brendel M Palleis C Beyer L Höglinger GU Schuh E. PET imaging of astrogliosis and tau facilitates diagnosis of Parkinsonian syndromes. Front Aging Neurosci. (2019) 11:249. 10.3389/fnagi.2019.00249 31572166 PMC6749151

[51] Takami Y Yamamoto Y Norikane T Mitamura K Hatakeyama T Nishiyama Y. ^18^F-THK5351 PET can identify lesions of acute traumatic brain injury. Clin Nucl Med. (2020) 45:e491–2. 10.1097/RLU.0000000000003165 32657863

[52] Charidimou A Perosa V Frosch MP Scherlek AA Greenberg SM van Veluw SJ. Neuropathological correlates of cortical superficial siderosis in cerebral amyloid angiopathy. Brain. (2020) 143:3343–51. 10.1093/brain/awaa266 32935842 PMC8453293

[53] Boyle PA Yu L Wilson RS Leurgans SE Schneider JA Bennett DA. Person-specific contribution of neuropathologies to cognitive loss in old age. Ann Neurol. (2018) 83:74–83. 10.1002/ana.25123 29244218 PMC5876116

[54] Martínez-Lizana E Carmona-Iragui M Alcolea D Gómez-Choco M Vilaplana E Sánchez-Saudinós MB. Cerebral amyloid angiopathy-related atraumatic convexal subarachnoid hemorrhage: an ARIA before the tsunami. J Cereb Blood Flow Metab. (2015) 35:710–7. 10.1038/jcbfm.2015.25 25735919 PMC4420868

[55] Catak C Zedde M Malik R Janowitz D Soric V Seegerer A. Decreased CSF levels of ß-amyloid in patients with cortical superficial siderosis. Front Neurol. (2019) 10:439. 10.3389/fneur.2019.00439 31105644 PMC6498501

[56] Vinters HV. Emerging concepts in Alzheimer's disease. Annu Rev Pathol. (2015) 10:291–319. 10.1146/annurev-pathol-020712-163927 25387055

[57] van Etten ES Verbeek MM van der Grond J Zielman R van Rooden S van Zwet EW. β-Amyloid in CSF: biomarker for preclinical cerebral amyloid angiopathy. Neurology. (2017) 88:169–76. 10.1212/WNL.0000000000003486 27903811 PMC5224714

[58] Shams S Granberg T Martola J Li X Shams M Fereshtehnejad SM. Cerebrospinal fluid profiles with increasing number of cerebral microbleeds in a continuum of cognitive impairment. J Cereb Blood Flow Metab. (2016) 36:621–8. 10.1177/0271678X15606141 26661151 PMC4794093

[59] Viswanathan A Greenberg SM. Cerebral amyloid angiopathy in the elderly. Ann Neurol. (2011) 70:871–80. 10.1002/ana.22516 22190361 PMC4004372

[60] Goldberg TE Huey ED Devanand DP Associations of APOE e2 genotype with cerebrovascular pathology: a postmortem study of 1275 brains. J Neurol Neurosurg Psychiatry. (2021) 92:7–11. 10.1136/jnnp-2020-323746 PMC11299059 33148816

[61] Yilmaz P Ikram MA Ikram MK Niessen WJ Viswanathan A Charidimou A. Application of an imaging-based sum score for cerebral amyloid angiopathy to the general population: risk of major neurological diseases and mortality. Front Neurol. (2019) 10:1276. 10.3389/fneur.2019.01276 31866930 PMC6908500

[62] An SJ Kim TJ Yoon BW. Epidemiology, risk factors, and clinical features of intracerebral hemorrhage: an update. J Stroke. (2017) 19:3–10. 10.5853/jos.2016.00864 28178408 PMC5307940

[63] Mustanoja S Strbian D Putaala J Meretoja A Curtze S Haapaniemi E. Association of prestroke statin use and lipid levels with outcome of intracerebral hemorrhage. Stroke. (2013) 44:2330–2. 10.1161/STROKEAHA.113.001829 23760210

[64] Li Z Shue F Zhao N Shinohara M Bu G. APOE2: protective mechanism and therapeutic implications for Alzheimer's disease. Mol Neurodegener. (2020) 15:63. 10.1186/s13024-020-00413-4 33148290 PMC7640652

